# Light-absorbing impurities in glacial environments over western Himalaya from reanalysis data and in situ observations

**DOI:** 10.1016/j.dib.2024.110602

**Published:** 2024-06-08

**Authors:** Imtiyaz Ahmad Bhat, Nadeem Ahmad Najar, Syed Danish Rafiq Kashani, Faisal Zahoor Jan, Irfan Rashid, Shahid Younis Bhat

**Affiliations:** Department of Geoinformatics, University of Kashmir, Hazratbal Srinagar 190006, Jammu and Kashmir, India

**Keywords:** MERRA-2, Snow-ice chemistry, Air-mass trajectories, Himalaya

## Abstract

In context with the scientific evidence of aerosol deposition induced snow and glacier melt, this paper provides baseline information about the spatiotemporal variability of aerosols and snow-ice chemistry filling the data and knowledge gap over the western Himalaya, India based on recently published paper [1]. A systematic approach was employed that entailed analysis of aerosol variability over four decades using MERRA-2 (Modern-Era Retrospective analysis for Research and Applications) data over five major mountain ranges in the western Himalaya. Further, data about nine physicochemical parameters was generated over three selected glaciers in the study area. HYSPLIT (HYbrid Single Particle Lagrangian Integrated Trajectory) model simulated air mass sources at weekly intervals. This dataset is valuable for future investigations aimed at understanding and characterizing the impacts of light-absorbing impurities on radiative forcing, albedo changes, snow-melt, glacier recession and water quality in the western Himalaya.

Specifications Table

**Table:** 

Subject	Earth and Planetary Sciences
Specific subject areas	Atmospheric chemistry; Geochemistry; Air pollution modelling.
Data format	Raw, processed and analyzed.
Type of data	Image: GeoTIFF, NetCDF, TIF Vector: SHP Text: TXT
Data collection	The MERRA-2 data with a spatial resolution of 0.625° × 0.5° were used. The data is available at https://disc.gsfc.nasa.gov/. The HYSPLIT model, available at https://www.arl.noaa.gov/hysplit/, was used to generate the local, regional and global aerosol sources on the three selected glaciers in the study area. ASTER DEM, available at https://search.earthdata.nasa.gov/search/, with a spatial resolution of 30 m was used for ascertaining the mean elevation of five mountain ranges. Total population was derived from the 1-km Gridded Population of the World (Version 4) dataset accessible from https://sedac.ciesin.columbia.edu/data/collection/gpw-v4.
Data source location	Name of the study area: Western Himalaya Mountain ranges: Pir Panjal range of Kashmir (PP), Greater Himalayan range of Kashmir (GH), Trans-Himalayan Zanskar range (ZA), Trans-Himalayan Ladakh range (LA), and Karakoram range (KA). Glaciers: Kolahoi, Machoi and Nehnar Affiliation: University of Kashmir, Hazratbal Srinagar Jammu and Kashmir, India Country: India Latitude: 32°17′ N and 37°6′ N Longitude:73°26′ E and 80°30′ E
Data accessibility	Repository name: Zenodo Data identification number: 10.5281/zenodo.10444504 Direct URL to data: https://zenodo.org/records/10444504
Related research article	I. Rashid, I. A. Bhat, N.A. Najar, S. Kang, F. Z. Jan, S. A. Dar, S. U. Bhat, S. D. R. Kashani, W. Rasool, Aerosol variability and glacial chemistry over the western Himalayas, Environ. Chem. 19 (2022) 312-327 https://doi.org/10.1071/EN22022

## 1 Value of the Data

• The dataset serves as a baseline for undertaking detailed aerosol-glacier investigations in the data-scarce Himalaya to precisely understand the influence of light-absorbing impurities on glacier melt besides climate change.
• The modelled air mass trajectories provide local, regional, and global sources of light-absorbing impurities and aerosols over western Himalaya that could be used by researchers for ascertaining the contribution of pollutants from Western Disturbances and Indian Summer Monsoons operating over the region.
• The physicochemical data of surface snow and ice samples on the selected glaciers could not only be used to develop satellite-based products pertaining to snow-ice chemistry but also be crucial in understanding the evolution of cryoconite holes and glacier-wide ice melt regimes.
• The data could be outscaled to other mountain ranges for initiating long term glaciohydrological research.

## 2 Background

The dataset compilation is crucial to understand the impact of aerosol variability and deposition on glacier melt in the western Himalaya, where such information was previously lacking. The study aimed to bridge this gap by analyzing aerosol variability using the MERRA-2 reanalysis data over 4 decades across five topographically distinct mountain ranges of the study area. Moreover, nine physicochemical parameters characterizing surface snow and ice of three glaciers in Greater Himalayan Mountain range were assessed to correlate with aerosol deposition.

## 3 Data Description

The aerosol variability was quantified using MERRA-2 reanalysis data across the five different mountain ranges, PP, GH, LA, KA, and ZA, in the western Himalaya. HYSPLIT model trajectories provide the local, regional and global sources of aerosols between December 2020 and November 2021. Physicochemical characteristics include pH, electrical conductivity (µS cm^−1^), TDS (mg L^−1^), TSS (mg L^−1^), TS (mg L^−1^), salinity (mg L^−1^), calcium (mg L^−1^), sulphate (mg L^−1^), and total phosphorous (µg L^−1^), of snow and ice samples of Kolahoi, Machoi and Nehnar glaciers. MERRA-2 data were used to assess the annual and seasonal aerosol variability of six variables Dust Column Mass Density (DUCMASS), Dust Surface Mass Concentration (DUSMASS), Dust Column Mass Density-PM 2.5 (DUCMASS2.5), Dust Surface Mass Concentration-PM 2.5 (DUSMASS2.5), Black Carbon Column Mass Density (BCCMASS) and Black Carbon Surface Mass Concentration (BCSMASS) across the five different mountain ranges. Studies over the region suggest that MERRA-2 data can be used to reveal the black carbon concentration in the region [[2], [3], [4]]. These data are provided in the Zenodo repository (raw.rar containing NetCDF files, processed.rar containing GeoTIFF files, seasonal.rar as Microsoft Excel files, annual.rar as Microsoft Excel files and figures.rar as TIF files). The MERRA-2 data extracted for the five different mountain ranges are provided as grid.rar containing SHP files. The characterization (climate pattern, population, mean elevation, temperature and participation) of the mountains ranges in the study area is provided as characterization.docx file. The HYSPLIT model trajectories, to identify the local, regional and global sources of aerosols, are stored as SHP (trajectories.rar) and TIF files (trajectories_map.rar). The comparison of physicochemical data of glacier ice and snow from this study with other regions of the Himalaya (from previous studies) is stored as comparasion.docx and physicochemical_figure.rar (TIF) files. The outlines of selected glaciers and sampling points of the snow and ice are provided as sample.rar. The total population data was derived from the 1 km Gridded Population of the World (Version 4) stored as gpw_v4.rar (GeoTIFF). The DEM (GeoTIFF), Landsat 8 OLI (GeoTIFF) and the study area (SHP) are provided as studyarea.rar and map.rar (TIF).

## 4 Experimental Design, Materials and Methods

The study utilized MERRA-2 reanalysis data spanning four decades from 1980 to 2020, obtained from NASAʼs Global Modelling and Assimilation Office (GOES-5) [5]. The ESRI ArcMap 10.6 facilitated the analysis of six key aerosol variables. The monthly data were used to produce seasonal and annual aerosol variability trends for the study area. The aerosol sources in glacial environments were identified using HYSPLIT model through back trajectories of air masses. HYSPLIT utilizes weekly Global Data Assimilation System (GDAS) meteorological data at 1°×1° spatial resolution provided by National Centre for Environmental Prediction (NCEP) [6]. The trajectory data, retrieved as SHP files from the HYSPLIT-WEB model, were analyzed seasonally. The model has been extensively used to track aerosol and moisture sources in mountain regions recently [[7], [8], [9]]. Snow and ice samples that were collected from glaciers were examined for 9 physicochemical parameters using standard protocols [10].

## Limitations

MERRA-2 is a coarse-resolution data (0.625°×0.5°) available at a monthly timestep. The monthly temporal resolution of the data might not capture short-term events operating at sub-daily, daily, weekly and sub-monthly intervals. Although the coarse spatial resolution of the MERRA-2 might not represent the spatiotemporal patterns of aerosols at glacier scale, it can be representative of regional aerosol variability. Further, MERRA-2 cannot resolve small-scale sources like local forest fires, biomass burning, industrial and automobile emissions that might be important for understanding aerosol-glacier interactions. While 3 glaciers have been chosen for geochemical analysis, a spatially distributed network of benchmark glaciers across Himalaya would offer better insights into local and regional impacts of aerosol deposition on glacier melt. These constraints highlight the necessity of employing more robust spatial datasets to capture local effects in the topographically complex Himalaya.

## Ethics Statement

No human or animal studies are presented in the manuscript. The study does not involve any data that was acquired from any social media platforms.

## CRediT authorship contribution statement

**Imtiyaz Ahmad Bhat:** Writing – original draft, Conceptualization, Methodology. **Nadeem Ahmad Najar:** Data curation, Methodology, Writing – review & editing. **Syed Danish Rafiq Kashani:** Visualization, Investigation, Writing – review & editing. **Faisal Zahoor Jan:** Visualization, Investigation, Writing – review & editing. **Irfan Rashid:** Writing – original draft, Supervision, Conceptualization, Writing – review & editing. **Shahid Younis Bhat:** Visualization, Writing – review & editing.

## Data Availability

Data for: Light-absorbing impurities in glacial environments over western Himalaya from reanalysis data and in situ observations (Original data) (Zenodo).
